# Trade Unionism: What Its Absence Permits

**Published:** 1913-09-13

**Authors:** 


### THE NATIONAL MEDICAL GUILD.

President: CHAS. BUTTAR, M.A., M.D., B.C.

Gen. Sec.: P. C. RAIMENT, M.R.C.S., L.R.C.P.

Offices: 34 Villiers Street, Strand, London, W.C.

### TRADE UNIONISM: WHAT ITS ABSENCE PERMITS.

The controversy that has been carried on in these columns during the last few weeks clearly demonstrates the necessity for the continued education of the profession. The letters of Dr. Fothergill were undoubtedly written, if he will allow me to say so, not in the spirit of inquiry, but actuated by the desire to ask the leaders of the trade union movement some awkward questions. How far the attempt was successful we must leave to the readers of this Journal, but before leaving the subject finally there are a few remarks to be made that the correspondence has suggested.

In the first place, the whole of Dr. Fothergill's questions could have been reduced to two in number —first, how can the profession be persuaded to join the union? and, secondly, how can they be made to obey the dictates of its council? The answer to each was fully discussed in the reply that the Guild made in the issue of August 23, and we do not propose to labour the subject, but we would like to suggest to readers who want to be informed as to the methods of commencing and holding together the members of any trade organisation that they read the account of the formation of the Pilots Union in Mark Twain's "Life on the Mississippi." It is true that the subject is burlesqued in Mark Twain's best method, but there are many grains of truth amongst the chaff.

The other contributor to the controversy, Dr. Harding Tomkins, rather took us to task about our criticism of the proposals of the British Medical Association. We appear to have raised a bigger storm than we intended, but we are quite unrepentant, and can only hope that the members of the Association are commencing to appreciate the fallacies of the scheme.

Our chief sin seems to have been that we stated that it would cost thirteen shillings and fourpence to collect a sovereign. It is quite true, as both Drs. Tomkins and Fothergill are so anxious to point out to us, that a portion of this money is to be expended for the benefit of the profession. That we will gladly concede, but at the same time we would like to mention that the Association contemplates raising its subscription to £2 2s. per annum, an advance of seventeen shillings per member. This on a basis of 20,000 members represents an increase in the annual income of £17,000, and in view of that we do ask whether it would not be possible to allocate the whole of the supposed fifty thousand to a reserve for the benefit of the profession. At the same time, even if the committee did that, we should still oppose the matter, because the fund would be liable to attachment, and at the risk of wearying our readers we must reiterate the warning given them by their own legal advisers in the report of council. They were distinctly warned there that no fund, *save that raised by a trade union is safe*. Surely in face of that warning the Association will never persist in its attempt to raise funds that might be of no use to the persons who had been instrumental in providing them.

Dr. Tomkins also takes us to task for what he calls "carping criticism." We are extremely sorry if we have overstepped the line of fair criticism of what is a matter of public interest, and we can assure Dr. Tomkins that nothing would give us greater pleasure than to see the Association adopt trade-union protection. Until they see fit to do so, as members of the Association we are at liberty to criticise any scheme that they may bring forward.

### The Debacle at Blackpool.

Tucked away in a few lines of print in the issue of the *British Medical Journal* Supplement, under date August 30, is a record of another tragedy of the medical profession. On August 18 the local medical committee and the panel practitioners of Blackpool met, and were interviewed by a certain Mr. Vivian sent to see them on behalf of the Commissioners. It will be remembered that the men of Blackpool declined to have any dealings with the "green vouchers" of "temporary residents," and on April 30 last they passed a resolution to that effect. After some discussion Mr. Vivian stated that if the members of the panel refused the terms offered the panel would be declared inadequate, and advantage taken of Section 11 of the new Act. (This means that whole-time officers would be appointed.) The next chapter was written on August 22 when the committee and the members of the panel again met, and by nine votes to six decided to refuse the terms laid down by the Commissioners.

The third and final meeting was held on August 24, when at an emergency meeting it was announced that three medical men had signified their willingness to accept the Commissioners' terms, and a telegram was read from the Commissioners that they were prepared to send to the town two medical men to attend to the temporary residents if necessary. The meeting therefore decided, by nine votes to seven, to abandon the attitude they had previously taken up and to advise the members of the panel to accept the "green vouchers" if they so wished.

A more melancholy record we do not think that it has been our lot to read since the great landslide of the early part of this year, and we refer to it because we want the whole profession to know that the Government have not ceased to stoop to the methods of the bludgeon and the press-gang in order to obtain their due proportion of slaves; and also because we desire that the profession should understand that unless they are prepared to make a stand somewhere there are no limits to the impositions which the Commissioners may lay upon their backs. It is difficult to understand what the men of Blackpool are thinking of, to give in thus tamely, and it makes the blood of every decent man boil to think that a great profession should be sold into slavery at the bidding of any Government. Surely if the facts were properly presented and given due publicity the profession would have responded to any call to help these men resist what they felt was an unjust demand. Now that these two hypothetical blacklegs have done their work, we presume that they will be added to that stage army of unattached medical men that exist solely in the fertile imagination of the Chancellor, and we do hope that if the same intimidation is tried in any other seaside resort, that the men will not be found so pliable, and, further, we hope that if they are attacked that they will apply to the Guild for help. We will gladly subscribe to the utmost of our ability to help them to stand firm, and we are sure that an appeal will bring the profession to their help. The spectacle of the profession being intimidated by two non-existent blacklegs is too humiliating to be endured a second time. And further, if the thing is not stopped, where is it going to end? Already the profession is having deductions made from its scanty pay, and, despite the assertion of Ministers to the contrary, that pay is in many cases in arrear. In the name of fortune what next? Will the profession never learn to be united, and from an isolated band of individualistic units rise to a strength that no Government dare tilt at.

The autumn campaign of the Guild will commence on October 15, and meetings will be held right up to the middle of December in all the principal towns of England, Scotland, and Wales. It is hoped by this means materially to increase the membership, and what is of more importance to bring the principles of trade unionism before a greater number of the profession. Should anyone reading these lines desire to assist by holding a meeting in his own neighbourhood, will he communicate with the General Secretary, at 34 Villiers Street, Strand? A full list of engagements will be published as soon as they are complete.

The next meeting of the Council of the Guild takes place at the Offices on Tuesday next, September 16, at 4 P.M. Amongst the business is the election of a new member of the Council, vice Dr. Ward resigned. P. C. R.

